# Neuroimmune Mechanisms as Novel Treatment Targets for Substance Use Disorders and Associated Comorbidities

**DOI:** 10.3389/fnins.2021.650785

**Published:** 2021-04-15

**Authors:** Mark D. Namba, Jonna M. Leyrer-Jackson, Erin K. Nagy, M. Foster Olive, Janet L. Neisewander

**Affiliations:** ^1^School of Life Sciences, Arizona State University, Tempe, AZ, United States; ^2^Department of Psychology, Arizona State University, Tempe, AZ, United States

**Keywords:** neuroinflammation, addiction, PTSD, MDD, chronic pain, HIV

## Abstract

Recent studies examining the neurobiology of substance abuse have revealed a significant role of neuroimmune signaling as a mechanism through which drugs of abuse induce aberrant changes in synaptic plasticity and contribute to substance abuse-related behaviors. Immune signaling within the brain and the periphery critically regulates homeostasis of the nervous system. Perturbations in immune signaling can induce neuroinflammation or immunosuppression, which dysregulate nervous system function including neural processes associated with substance use disorders (SUDs). In this review, we discuss the literature that demonstrates a role of neuroimmune signaling in regulating learning, memory, and synaptic plasticity, emphasizing specific cytokine signaling within the central nervous system. We then highlight recent preclinical studies, within the last 5 years when possible, that have identified immune mechanisms within the brain and the periphery associated with addiction-related behaviors. Findings thus far underscore the need for future investigations into the clinical potential of immunopharmacology as a novel approach toward treating SUDs. Considering the high prevalence rate of comorbidities among those with SUDs, we also discuss neuroimmune mechanisms of common comorbidities associated with SUDs and highlight potentially novel treatment targets for these comorbid conditions. We argue that immunopharmacology represents a novel frontier in the development of new pharmacotherapies that promote long-term abstinence from drug use and minimize the detrimental impact of SUD comorbidities on patient health and treatment outcomes.

## Introduction

Substance Use Disorders (SUDs) are a significant public health concern and remain a leading cause of preventable death in the United States (US) and worldwide. Moreover, the economic burden of SUDs is substantial, amounting to more than $740 billion annually within the US in health care costs, lost productivity, and crime (National Institute on Drug Abuse [NIDA], 2020d). Current FDA-approved pharmacotherapies used to treat SUDs primarily function to reduce withdrawal symptoms via partial receptor stimulation or to prevent drug reinforcement through receptor inhibition. Such strategies can reduce drug cravings and promote abstinence, yet relapse rates remain high even for individuals receiving such treatments (National Institute on Drug Abuse [NIDA], 2020b). Impediments to successful treatment include comorbidities such as depression, anxiety, and other physical ailments that are not adequately addressed by current treatment strategies (Swendsen et al., 2010). Thus, new and effective treatments that target shared mechanisms underlying these comorbidities may enhance treatment outcomes.

Decades of research have revealed distinct neural mechanisms involved in various phases of SUD development. Early work utilizing animal models focused on the effects of drug exposure on mesolimbic dopamine transmission originating from the ventral tegmental area (VTA) and terminating in corticolimbic structures such as the nucleus accumbens (see Koob and Volkow, 2010 for review). More recent studies have expounded the role of other molecular pathways and cellular circuitries that are involved in the cycle of addiction (Koob and Volkow, 2016). For example, the role of glial cells in modulating drug-induced neural plasticity and behavior has emerged as an important topic in the study of the pathophysiology of SUDs (Miguel-Hidalgo, 2009). Specifically, glia within the CNS and immune cells within the periphery are capable of modulating brain plasticity and behavior through complex interactions, and mounting evidence suggests that these interactions may underlie substance abuse and relapse vulnerability (Crews and Vetreno, 2011; Crews et al., 2011; Clark et al., 2013; Cui et al., 2014).

There are several recent and thorough reviews on neuroimmune mechanisms of SUDs (Cui et al., 2014; Bachtell et al., 2015; Jacobsen et al., 2016; Crews et al., 2017; Hofford et al., 2019) and other neuropsychiatric disorders (Hodes et al., 2015; Wohleb et al., 2016; Bekhbat and Neigh, 2018; Brenhouse et al., 2019). Here, we focus on literature from within the last 5 years when possible to highlight the most recent advances in neuroimmune mechanisms of SUDs across several common drugs of abuse. As well, we highlight recent studies on neuroimmune mechanisms of psychiatric and non-psychiatric comorbidities and discuss how these studies reveal intersections with the SUD literature. Specifically, we discuss the neuroimmune mechanisms that are associated with addiction-related processes, first focusing on neuron-glia interactions and how they underlie synaptic plasticity, learning, and memory with cytokine signaling as a case in point. Next, we highlight recent preclinical studies that have identified immune processes that are associated with drug-induced neural plasticity and behavior. We then discuss how recent studies have implicated neuroimmune mechanisms in the comorbidity of SUDs with other diseases and disorders, and we describe how targeting these underlying neuroimmune mechanisms may represent a novel approach toward improving treatment outcomes. Lastly, we provide recommendations for future studies that aim to identify and describe neuroimmune mechanisms underlying SUDs and associated comorbidities.

## Cytokines Critically Regulate Synaptic Plasticity, Learning, and Memory

**FIGURE 1:**
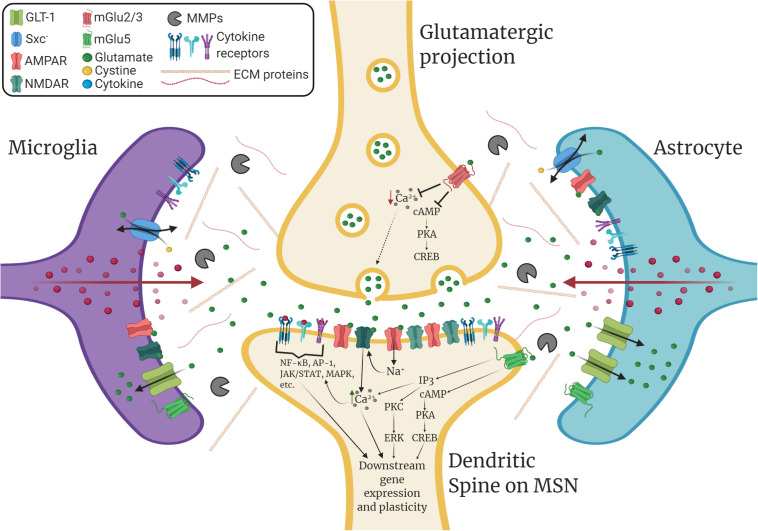
Immunomodulation of striatal glutamatergic synaptic plasticity. GABAergic medium spiny neurons (MSNs) within the nucleus accumbens (NAc) receive glutamatergic inputs from various corticolimbic structures that drive drug-seeking behavior, which includes the prelimbic cortex (PrL), the amygdala (AMY), and the hippocampus (HPC). Glutamatergic plasticity at dendritic spines on MSNs is regulated by glia and extracellular matrix signaling. Glial cell-derived neuroimmune signals such as cytokines play a significant role in modulating this plasticity. Additionally, glutamate transporters such as GLT-1 and the cystine-glutamate exchanger, system xc- (Sxc^–^), which are found on both astrocytes and microglia, tightly regulate extracellular levels of glutamate. Microglia and astrocytes express ionotropic and metabotropic glutamate receptors (e.g., mGlu5) and can release neuroimmune factors in response to glutamatergic stimulation. Within the extracellular matrix (ECM), enzymes such as matrix metalloproteinases (MMPs), which are predominantly produced by microglia and astrocytes, can remodel the ECM to facilitate dendritic spine plasticity. Cytokines can promote the expression and activation of MMPs, which are associated with learning, memory, and synaptic plasticity. Cytokines also bind directly to receptors located on both neurons and glia, which can directly influence downstream gene expression and synaptic plasticity. This can result in changes in glutamate transporter expression, glutamate receptor surface expression, intracellular signaling, gene expression, dendritic spine morphology, and post-synaptic excitability.

Glia play diverse roles in dynamically modulating synaptic plasticity, learning, and memory beyond their “traditional” roles in supporting tissue homeostasis (Temburni and Jacob, 2001; Ben Achour and Pascual, 2010; Perea et al., 2014). For example, neurochemical signaling molecules such as glutamate mediate neuron-glia crosstalk that can alter downstream immunomodulatory signaling. As illustrated in Figure 1, microglia, which continuously survey their environment with ramified processes, express both ionotropic and metabotropic glutamate receptors and can release proinflammatory factors in response to rapid changes in extracellular glutamate levels (Hagino et al., 2004; Murugan et al., 2011; Liu H. et al., 2016). Similarly, astrocytes express such receptors and dynamically respond to rapid changes in synaptic glutamate levels through modulation of glutamate uptake (Duan et al., 1999) and through gliotransmission via adenosine triphosphate (ATP) and other transmitters (Harada et al., 2016). As well, astrocytes are highly sensitive to immunomodulatory signals and this has indirect consequences on astrocytic regulation of synaptic transmission (Cekanaviciute and Buckwalter, 2016). Together, microglia and astrocytes can orchestrate potent modulatory control over synaptic plasticity through glial cell-derived immunomodulatory factors such as cytokines.

Cytokines have been extensively studied for their role in learning, memory, and synaptic plasticity (Levin and Godukhin, 2017), and the outcome of these processes depend on the specific cytokine, its concentration within the brain, receptors available for cytokine binding and activation of signal transduction pathways, and the conditions underlying cytokine release (Goshen and Yirmiya, 2007). For example, Beattie et al. (2002) and Stellwagen et al. (2005) have demonstrated that glial tumor necrosis factor alpha (TNFα) facilitates membrane insertion of calcium-permeable α-amino-3-hydroxy-5-methyl-4-isoxazolepropionic acid (AMPA) glutamate receptors (CP-AMPARs) and internalization of γ-aminobutyric acid type A (GABA_A_) receptors within the hippocampus, leading to enhanced excitatory synaptic transmission. In contrast, Lewitus et al. (2014) found that TNFα exerts an opposite effect at striatal synapses, where TNFα internalizes CP-AMPARs and reduces corticostriatal synaptic strength. These investigators demonstrated that microglia-induced TNFα release depresses excitatory synaptic activity within the ventral striatum through internalization of AMPARs and that this process is associated with cocaine-induced locomotor sensitization (Lewitus et al., 2016). Another example of region-specific regulation of synaptic plasticity showed that TNFα secretion in response to peripheral nerve injury enhances excitatory synaptic connectivity within the spinal cord but impairs this connectivity within the hippocampus (Liu et al., 2017). These studies provide clear evidence that immunomodulatory signals such as TNFα crucially regulate synaptic plasticity in a brain region-specific manner, which may have important implications for understanding the pathophysiology of SUDs.

Like TNFα, microglia and astrocytes also release interleukin-6 (IL-6) within the CNS, a cytokine involved in modulating learning and memory (Ye and Johnson, 1999; Dong and Benveniste, 2001; Choi et al., 2014). Early studies demonstrated that acute IL-6 exposure inhibits long-term potentiation (LTP) within the hippocampus likely through inhibition of mitogen-activated protein kinase/extracellular signal-regulated kinase (MAPK/ERK) signaling (Li et al., 1997; Tancredi et al., 2002). In addition, overexpression of IL-6 in astrocytes of mice results in reduced LTP in the dentate gyrus (Bellinger et al., 1995). However, *in vitro* and *in vivo* studies examining IL-6 expression during the induction of hippocampal LTP show that LTP induces an upregulation of IL-6 mRNA that is localized to non-neuronal cells such as astrocytes (Jankowsky et al., 2000; Balschun et al., 2004) and inhibition of IL-6 signaling results in improved performance in hippocampus-dependent memory tasks (del Rey et al., 2013). Consistent with this finding, IL-6 knockout mice exhibit enhanced performance in a radial arm maze task compared to wild type mice, which is correlated with hippocampal choline acetyltransferase activity (Braida et al., 2004).

Interleukin-1β (IL-1β) is also critically involved in hippocampal-dependent learning and synaptic plasticity. Within the hippocampus, fear conditioning and LTP upregulate IL-1β and systemic administration of small concentrations of IL-1β can enhance learning and memory (Schneider et al., 1998; Balschun et al., 2003; Goshen et al., 2007; del Rey et al., 2013). Additionally, acute intra-hippocampal administration and chronic overexpression of IL-1β can impair fear conditioning and spatial learning as well as hippocampal LTP similar to IL-6 (Vereker et al., 2000; Loscher et al., 2003; Ross et al., 2003; Gonzalez et al., 2009; Moore et al., 2009; Hein et al., 2010; MacHado et al., 2010). Despite these seemingly convergent and consistent findings, studies examining learning (e.g., fear conditioning) and hippocampal plasticity using IL-1 receptor (IL-1R) knockout mice have demonstrated mixed effects (Avital et al., 2003; Koo and Duman, 2009; Murray et al., 2013). This is likely the result of complex interactions with other cytokines within the IL-1 family, which is a very broad family of closely related cytokines (Dinarello, 2018).

These studies provide clear evidence that cytokines can exert a dynamic modulatory role within the nervous system over synaptic plasticity, learning, and memory. They also highlight that the specific cytokine and conditions in which neural circuits are exposed to them are paramount when interpreting these findings. This complexity is a primary reason why elucidating the role of immunomodulation and neuron-glia crosstalk in facilitating the formation and persistence of addiction-related behaviors remains challenging. Below we highlight recent preclinical studies, within the last 5 years when possible, that demonstrate the diverse nature of immunomodulation in regulating addiction-related behaviors and underscore some of the remaining gaps in our understanding of these processes.

## Recent Preclinical Studies Examining Immunomodulation of Addiction-Related Behaviors and Associated Neuroplasticity

Since 2015, hundreds of articles related to immune mechanisms involved in SUDs have been published, ranging from cellular and molecular studies exploring novel neuroimmune mechanisms of addiction-related behaviors to clinical studies examining the efficacy of immunopharmacology in the treatment of SUDs and associated comorbidities. For example, a PubMed search conducted at the time of preparation of this manuscript for all articles within the last 5 years containing the keywords “addiction” and “immune” yielded over 900 results, showing the exponential growth of research in this area. Of relevance to the current review, preclinical animal models of SUDs have proven invaluable in advancing our understanding of the social, psychological, and biological etiology of SUDs (Lynch et al., 2010; Kuhn et al., 2019), and recent studies utilizing these models have revealed valuable insights into the significant role neuroimmune mechanisms play in regulating drug reward and motivation. Here, we highlight key preclinical research developments in this area, focusing on cocaine, methamphetamine, nicotine, alcohol, and opioids. This review is by no means intended to be exhaustive, but rather serves to highlight recent advances, common themes, and lingering gaps within the literature geared to inform future investigations into the neuroimmune mechanisms of SUDs.

### Cocaine

Since 2015, cocaine overdose deaths, particularly those involving synthetic opioids, have risen sharply to over 15,000 as of 2019 in the United States (National Institute on Drug Abuse [NIDA], 2021), highlighting a resurgence of psychostimulant abuse in the wake of the opioid epidemic. Importantly, no effective medications exist that successfully treat cocaine use disorders (CUDs). Studies in humans have demonstrated that CUDs are associated with altered serum levels of pro- and anti-inflammatory cytokines (Moreira et al., 2016; Zaparte et al., 2019). As well, serum cytokine levels may be useful indicators for assessing the severity for CUDs and identifying effective treatment strategies, although sex differences and psychiatric comorbidity are important factors to consider (Araos et al., 2015; Pedraz et al., 2015; Maza-Quiroga et al., 2017; Pianca et al., 2017). Thus, preclinical investigation into the impact of cocaine on immune function may reveal novel targets for pharmacotherapy development. In a recent study by Calipari et al. (2018), both non-contingent cocaine exposure and cocaine self-administration in mice were found to upregulate serum levels of granulocyte-colony stimulating factor (G-CSF), a known growth factor regulator of granulocytes, which positively correlated with cocaine-induced locomotor sensitization. This study also found that both acute and sub-chronic cocaine exposure increased G-CSF mRNA expression within the nucleus accumbens (NAc) and medial prefrontal cortex (mPFC) and that G-CSF potentiates cocaine self-administration. This effect is likely not specific to cocaine, as this same group also demonstrated that G-CSF enhances sucrose motivation and cognitive flexibility, which they hypothesize may be through indirect modulation of mesolimbic dopamine transmission via other immunomodulators such as TNFα (Kutlu et al., 2018). As well, the role of G-CSF in cocaine-induced dopaminergic plasticity was also shown to be dependent on estrous cycle phase in female mice (Brady et al., 2019), suggesting important sex differences underlying immunomodulation of drug-induced neural and behavioral plasticity.

As mentioned above, TNFα is a critical modulator of synaptic plasticity and specifically cocaine-induced neuroadaptations. A recent study demonstrated that non-contingent cocaine exposure in mice activated microglia and increased TNFα in the NAc, leading to depressed excitatory synaptic strength at striatal synapses (Lewitus et al., 2016). Interestingly, this study showed that treatment with a weak agonist of toll-like receptor 4 (TLR4) resulted in microglia activation, reduced cocaine-induced locomotor sensitization, and decreased excitatory activity within the NAc. This study suggests that TNFα plays an adaptive role in suppressing cocaine-induced neurobehavioral plasticity. Nevertheless, the effects of TNFα on downstream cell signaling and subsequent changes in gene expression and cellular physiology are sensitive to the concentration and duration of exposure to these immunomodulators. For example, nuclear factor kappa B (NF-κB), which is a DNA-binding protein complex that is induced by TNFα, exhibits autoregulatory feedback and oscillatory nuclear translocation with prolonged upstream receptor stimulation. This allows cells to discriminate against multiple levels of cytokine input and subsequently fine-tune downstream gene transcription (Inoue et al., 2016; Zambrano et al., 2016; Zhang Q. et al., 2017). Moreover, the effects of immunomodulators such as TNFα are likely drug-specific and dependent on the preclinical behavioral model (e.g., see Araos et al., 2015 for comparison to nicotine). Indeed, attention to these considerations is important for understanding the role of neuroimmune signaling in the pathophysiology of SUDs. Recent studies examining mesolimbic neuroimmune signaling implicate proinflammatory signaling mechanisms as an important regulator of cocaine-induced neurobehavioral plasticity. For example, Brown et al. (2018) demonstrated that antagonism of TLR4 within the ventral tegmental area (VTA), which sends dopaminergic projections to corticolimbic structures such as the striatum to drive drug-seeking, reduces cocaine-primed reinstatement of drug seeking with no effect on sucrose seeking. As well, stimulation of the VTA with lipopolysaccharide (LPS), which activates TLR4, modestly reinstates cocaine seeking, and inhibition of cocaine-induced elevations in IL-1β within the VTA reduces cocaine seeking (Brown et al., 2018). This study corroborates a previous study demonstrating that cocaine binds uniquely with the TLR4 receptor complex within the VTA to modulate dopamine input into the NAc and subsequent cocaine-seeking behavior (Northcutt et al., 2015). TLR4 knockout mice exhibit attenuated cocaine conditioned place preference (CPP), which may be due to impairments in excitatory synaptic plasticity within the NAc core (Kashima and Grueter, 2017). These findings are consistent with the hypothesis that dopamine input into the NAc and cocaine-mediated activation of TLR4 stimulate microglial activation, resulting in TNFα release that serves as a feedback mechanism to scale down drug-induced increases in synaptic strength. Nevertheless, whether these neuroimmune mechanisms play a significant role in abstinence-dependent changes in mesocorticolimbic plasticity and subsequent relapse remains unclear.

Like cytokines, chemokines are immunomodulators that are influenced by cocaine and may be important regulators of cocaine-induced neurobehavioral plasticity. Traditionally, chemokines promote cellular chemotaxis and are important for directing both homeostatic and inflammatory immune responses. Kim et al. (2017) recently demonstrated that inhibiting the chemokine receptor CXCR4 with the antagonist AMD3100 reduces cocaine CPP and cocaine-induced locomotion. Similar effects are also observed with the synthetic cathinone methylenedioxypyrovalerone (MDPV) (Oliver et al., 2018), which mimics cocaine in its physiological and behavioral effects. Interestingly, this same group has recently provided the first evidence that the chemokine receptor CCR5, which is highly implicated in the pathophysiology of human immunodeficiency virus (HIV), is upregulated by cocaine and regulates the formation of cocaine CPP (Nayak et al., 2020). Cocaine is known to facilitate HIV invasion and viral replication within the brain (Zhang et al., 1998; Sahu et al., 2015; Tyagi et al., 2016) and HIV may also potentiate the reinforcing properties of cocaine (McIntosh et al., 2015). Thus, these studies highlight how neuroimmune mechanisms may underly the intersection between SUDs and other comorbidities, including non-psychiatric comorbidities such as HIV. As discussed below, other psychostimulants such as methamphetamine also exhibit similar qualities.

### Methamphetamine

Over the last decade, methamphetamine (METH) use has risen sharply, particularly among individuals that also use opioids. Specifically, the number of psychostimulant overdoses (consisting primarily of METH), both with and without opioids, has been sharply rising within the United States over the last decade in the wake of the opioid epidemic (Mattson et al., 2021). This highlights an important need for novel therapeutic strategies to treat psychostimulant use disorders, which currently have no FDA-approved pharmacological treatments. METH has a profound impact on immune system function through disruption of the blood–brain barrier, dysregulation of both central and peripheral immune signaling, epigenetic modifications, and perturbation to the gut microbiome (for a recent review, see Prakash et al., 2017). For instance, METH alters the expression of microRNAs (miRNAs) that regulate immune- and addiction-related genes (Zhu et al., 2015, 2016). A recent study demonstrated that METH exposure in mice downregulates miR-29b and miR-124 within the NAc, which is consistent with previous observations in animals treated with cocaine (Chandrasekar and Dreyer, 2009; Eipper-Mains et al., 2011; Zhu et al., 2016). These miRNAs are crucial regulators of immune system function (Liston et al., 2012; Qin et al., 2016), suggesting that METH (and possibly psychostimulants in general) may exert aberrant immunomodulatory effects over mesocorticolimbic reward pathway signaling through dysregulation of miRNA expression. In addition to indirect immunomodulation of drug-induced plasticity via miRNA regulation, METH interacts directly with immune cells to promote neuroinflammation and alter reward learning. Indeed, a recent report examining METH’s effects on TLR4 signaling and VTA-NAc dopamine transmission provides convincing evidence of this fact. In this study, Wang X. et al. (2019) report that METH can bind to lymphocyte antigen 96 (i.e., MD-2), which interacts directly with TLR4 to confer receptor responsiveness to LPS, and that inhibition of TLR4 attenuated METH-induced NF-κB activation in microglia. In addition, this study also showed that METH upregulates IL-6 within the VTA and that this is associated with enhanced extracellular dopamine within the NAc, and treatment with the TLR4 inhibitor (+) naloxone or an intra-VTA IL-6 antibody reduces METH-induced increases in NAc dopamine (Wang X. et al., 2019). This study parallels findings described above by Brown et al. (2018), which demonstrated reduced cocaine-primed reinstatement following intra-VTA treatment with a TLR4 antagonist. These findings collectively suggest that psychostimulants such as METH and cocaine may alter dopamine transmission and reward processing through similar immunomodulatory mechanisms such as TLR4 signaling.

Another recent study examining the potential role of dopamine D1 receptors (D1Rs) in mediating METH’s immunomodulatory effects found that acute METH exposure increases LPS-induced IL-6 and TNFα release within the NAc, hippocampus (HPC), and caudate-putamen (CPu), and that systemic inhibition of D1Rs suppresses this effect (Wang B. et al., 2019). This study suggests that METH primes neuroimmune responses to inflammatory stimuli within the mesocorticolimbic reward circuitry and that METH-induced dopamine transmission may be involved in this process. Nevertheless, whether these processes confer increased susceptibility to METH relapse remains unclear. An intriguing pair of studies has attempted to address this gap within the literature, where investigators used a CPP paradigm to probe whether cannabidiol (CBD) reduces reinstatement of METH seeking in animals that experienced sleep deprivation and if CBD modulates cytokine expression within the prefrontal cortex (PFC) and HPC (Karimi-Haghighi and Haghparast, 2018; Karimi-Haghighi et al., 2020). These studies collectively showed that METH-induced reinstatement is accompanied by increased expression of TNFα and the anti-inflammatory cytokine IL-10 within the PFC and HPC, and CBD treatment significantly reduces reinstatement as well as proinflammatory cytokine levels within these brain regions. Interestingly, CBD treatment before sleep deprivation elevated TNFα, IL-1β, IL-6, and IL-10 levels within the HPC but reduced IL-10 levels within the PFC. These results indicate that CBD may attenuate METH-seeking behavior through immunomodulatory mechanisms. However, the clear heterogeneity of cytokine expression profiles across the brain raise important questions regarding the functional role of these cytokines within specific neural circuits. Thus, further studies that attempt to dissect possible circuit-specific mechanisms of immunomodulation are warranted.

One limitation of the studies above is that METH was experimenter-delivered to the animals, as opposed to self-administered. Previous studies demonstrate that the magnitude of METH effects on the CNS, such as on dopamine transporter (DAT) expression, is larger in experimenter-delivered METH exposure models as compared to human clinical and post-mortem studies (Wilson et al., 1996; Volkow et al., 2001a, b; Sekine et al., 2003). Indeed, self-administration models better recapitulate the effects of METH on striatal DAT function and expression observed in humans (McFadden et al., 2012). As well, experimenter-delivered METH models do not allow investigators to correlate drug-induced neuroplasticity with behavioral measures such as escalation of drug intake and cue-motivated drug seeking. Thus, self-administration models examining the neuroimmune consequences of chronic METH use provide a clearer understanding of how these immune mechanisms facilitate METH abuse and relapse vulnerability in humans.

Recent investigations using self-administration paradigms implicate neuroinflammation and disruption of blood-brain barrier (BBB) integrity as a consequences of chronic METH use. For example, Gonçalves et al. (2017) examined the impact of extended-access METH self-administration and abstinence on BBB integrity and neuroinflammation within the HPC and striatum. This study found that METH self-administration and abstinence downregulated tight junction proteins and collagen IV, indicating reduced BBB integrity, as well as astrogliosis, microgliosis, and upregulated proinflammatory mediators such as TNFα, IL-1β, and matrix metalloproteinase-9 (MMP-9) (Gonçalves et al., 2017). While this study did not examine whether these METH-induced neuroadaptations are directly involved in METH-seeking behaviors, many other studies discussed previously have implicated TNFα and IL-1β in regulating learning, memory, and synaptic plasticity, and MMP-9 in particular has also been implicated in these processes as well as in cue-motivated drug seeking specifically (Mizoguchi et al., 2007; Knapska and Kaczmarek, 2015; Smith et al., 2015). METH-induced impairments on the BBB are well documented, and the consequences of these proinflammatory effects of METH on the BBB can exacerbate invasion of viruses as well as peripheral immune cells into the brain (see Northrop and Yamamoto, 2015 for review). Indeed, individuals who use METH are significantly more vulnerable to HIV and the neurocognitive dysfunctions associated with chronic HIV infection (Borgmann and Ghorpade, 2015; Kesby et al., 2015a). Recent preclinical animal studies demonstrate that HIV and its proteins exacerbate METH-induced deficits in learning and memory (Hoefer et al., 2015; Kesby et al., 2015a, b) as well as METH reward sensitivity and behavioral sensitization (Liu et al., 2009; Kesby et al., 2014). Taken together, METH produces significant perturbations to cellular systems within the mesocorticolimbic reward system through immunomodulation and, akin to cocaine, these immunomodulatory mechanisms may underlie the co-morbidity of chronic METH use and both psychiatric and non-psychiatric diseases.

### Nicotine

Unlike other drugs of abuse such as alcohol, cocaine, and methamphetamine, relatively little preclinical research exists on the role of immunomodulation in nicotine addiction-related behaviors. Previous work demonstrates that nicotine produces predominantly anti-inflammatory and pro-cognitive effects, which has been largely attributed to nicotine’s full-agonist activity at α7-containing nicotinic acetylcholine receptors (Kalra et al., 2004; Shytle et al., 2004; Foucault-Fruchard and Antier, 2017) (nAChRs; although see Thomsen and Mikkelsen, 2012). For example, recent studies suggest that nicotine’s acute anti-inflammatory effects via α7-containing nAChR signaling may improve learning and memory and reduce the severity of neurocognitive symptoms associated with neurodegenerative diseases such as Alzheimer’s disease. Specifically, a recent study demonstrated that nicotine attenuates LPS-induced neuroinflammation within the HPC and associated cognitive deficits in spatial learning (Wei et al., 2015). As well, another study showed that inhibition of α7-containing nAChRs with an α7-specific antibody is sufficient to produce neuroinflammation, accumulation of β-amyloid, and memory impairments in mice (Lykhmus et al., 2015). While these studies suggest that nicotine may be therapeutic within certain clinical contexts (e.g., Alzheimer’s disease), there are a number of caveats to consider. Firstly, individuals who are dependent on nicotine largely smoke tobacco, which can produce profoundly different effects on cognition and neuroinflammation when compared to nicotine alone (Swan and Lessov-Schlaggar, 2007). This complicates the interpretations one can make from preclinical nicotine studies regarding the neuroimmune and neurocognitive effects of smoking in humans. Additionally, acute versus chronic nicotine, as well as varying withdrawal periods, are all associated with significant differences in whether they are associated with neuroinflammation and cognitive deficits. For example, a recent study revealed cognitive impairment and neuroinflammation within the PFC and HPC of mice 4 days after mecamylamine-precipitated nicotine withdrawal, which was reversed by cannabidiol (Saravia et al., 2019). This contrasts the studies described above demonstrating anti-inflammatory and cognition-enhancing effects of acute or sub-chronic nicotine treatment in inflammatory disease models. Furthermore, these disparate findings in the literature may be attributed to the heterogenous distribution of nAChRs throughout the CNS (Gotti et al., 2009). Given the significant role of nAChRs in immune system function [particularly the α7-containing nAChR (Fujii et al., 2017)], the heterogeneity of nAChR distribution within the CNS could have significant implications for immunomodulation of nicotine addiction-related behaviors.

Several recent studies have attempted to address these significant gaps in the literature by investigating whether neuroimmune mechanisms within mesocorticolimbic reward circuitry are involved in nicotine addiction-related behaviors. In our recent study, we attempted to investigate whether changes in NAc core neuroimmune signaling were associated with nicotine self-administration, extinction, and cue-induced reinstatement as well as the therapeutic efficacy of the antioxidant compound *N*-acetylcysteine (NAC). NAC, which has been used traditionally as a mucolytic and as a treatment for acetaminophen overdose, is known to reverse drug-induced perturbations in glutamatergic homeostasis within the NAc and to inhibit cue-motivated drug seeking (Baker et al., 2003; Moran et al., 2005; Moussawi et al., 2009; Deepmala et al., 2015; Reissner et al., 2015; Elbini Dhouib et al., 2016; Powell et al., 2019). We demonstrated that 2 weeks of extinction training following nicotine self-administration was associated with enhanced TNFα expression within the NAc core and concomitant downregulation of the astrocytic glutamate transporter GLT-1. As well, we showed that NAC ameliorated these deficits and blocked cue-induced reinstatement of nicotine seeking through GLT-1- and NF-κB-dependent mechanisms within the NAc core (Namba et al., 2019). Unlike TNFα, IL-6 was not upregulated after a period of extinction and cue reinstatement testing, although glial fibrillary acidic protein (GFAP) expression was downregulated at these timepoints. No changes in TNFα and GFAP expression were observed immediately following nicotine self-administration when compared to yoked saline controls. This is consistent with a previous report showing downregulated NAc GFAP expression following 2 weeks of cocaine extinction (Scofield et al., 2016). Our findings were also corroborated by another recent study demonstrating that withdrawal from experimenter-delivered nicotine exposure in mice upregulates TNFα and IL-1β mRNA within the NAc and increases anxiety-like behavior, both of which are prevented by pharmacological depletion of microglia with a colony-stimulating factor 1 receptor inhibitor (Adeluyi et al., 2019). Moreover, another recent study showed that co-administration of the non-steroidal anti-inflammatory drug acetylsalicylic acid with NAC significantly attenuates oral nicotine self-administration as well as consumption of nicotine following a period of nicotine deprivation (Quintanilla et al., 2021).

Taken together, the above findings highlight the need for future studies to evaluate multiple timepoints when attempting to describe neuroimmune mechanisms of addiction-related behaviors. As well, these data underscore the need for investigators to exhibit caution when describing their findings related to the neuroimmune consequences of nicotine exposure. While it can be tempting to ascribe labels such as “neuroinflammation” to processes that upregulate proinflammatory cytokines such as TNFα, the studies above illustrate that these immunomodulators exhibit diverse functions that serve to fine-tune neuronal plasticity and behavior, and dysregulation of a specific cytokine may not be indicative of a net “inflammatory” or “anti-inflammatory” process *per se*. Significant gaps remain in the understanding of whether nicotine alters neuroimmune signaling within the brain’s mesocorticolimbic reward circuitry and whether such changes are relevant to the reinforcing and incentive motivational properties of nicotine. However, α7-containing nAChRs are critically involved in nicotine addiction-related behaviors, and it is possible that neuroimmune mechanisms downstream of α7-containing nAChRs mediate the modulatory role of this receptor type over nicotine reward and reinforcement.

The α7 nAChR subunit plays a critical yet complex role in mediating the effects of nicotine on immune system function. For example, a recent study demonstrated that an α7-containing nAChR agonist inhibits LPS-induced astroglial release of proinflammatory cytokines and NF-κB activation *in vitro* (Patel et al., 2017). Moreover, another recent study showed that α7-containing nAChR agonist treatment improves neuronal survival and reduces microglial activation in rats treated with an excitotoxic lesion within the striatum (Foucault-Fruchard et al., 2017). While many studies demonstrate an anti-inflammatory and protective role of α7-containing nAChR stimulation in a variety of disease models, other studies offer conflicting conclusions (Thomsen and Mikkelsen, 2012). In addition to its role in regulating neuroimmune signaling, the α7 nAChR subunit has also been implicated in the reinforcing properties of nicotine (Levin et al., 2009; Besson et al., 2012; Brunzell and McIntosh, 2012). In a recent study examining nicotine-induced changes in α7 expression within the striatum and frontal cortex of mice, experimenter-delivered, nicotine-containing electronic cigarette vapor exposure upregulated α7 expression within these brain regions (Alasmari et al., 2017). In contrast, another recent study demonstrated that nicotine consumption in rats is negatively correlated with α7 expression within the HPC, which is most prominent in females (Gozen et al., 2016). Indeed, the heterogeneity of nAChR expression throughout the brain as well as the differential neurobehavioral impact of experimenter-delivered drug exposure versus self-administration likely contributed to these seemingly discrepant findings (Gotti et al., 2009; Namba et al., 2018). Additionally, such differences may suggest that nicotine alters α7-containing nAChR expression differentially throughout the brain, which could produce brain region-specific differences in downstream neuroimmune signaling. Varenicline, which is a popular smoking-cessation medication, has been reported to be a full agonist at α7-containing nAChRs in addition to a partial agonist at α4β2^∗^ nAChRs (Mihalak et al., 2006). Interestingly, a recent study demonstrated that combination therapy of varenicline with the weak monoamine reuptake inhibitor bupropion reduced nicotine self-administration in rats (Hall et al., 2015), and bupropion has been shown to exhibit anti-inflammatory properties in mice (Hajhashemi and Khanjani, 2014). However, another recent study concluded that discontinuation of varenicline treatment may increase relapse vulnerability. Specifically, this study showed that rats receiving varenicline treatment exhibited impaired extinction learning, which was particularly evident in varenicline-treated rats that underwent a second cycle of nicotine self-administration and extinction training without continued varenicline treatment (Macnamara et al., 2016). Perturbations to α7-containing nAChRs and subsequent α7-mediated neuroimmune responses may also come from flavor additives in tobacco products such as menthol, which has been shown to inhibit α7-containing nAChR receptor function (Ashoor et al., 2013) and facilitate nicotine self-administration (Biswas et al., 2016). Ultimately, it remains unclear whether immunomodulatory mechanisms, such as those elicited by α7-containing nAChRs, are relevant to the formation and persistence of nicotine addiction-related behaviors. As well, it is unclear if such mechanisms would be effective targets for medications development. While recent preclinical studies have attempted to shed light on this gap in the literature (Adeluyi et al., 2019; Namba et al., 2019), future research that utilizes preclinical models of volitional drug self-administration, withdrawal, and cue-motivated nicotine seeking is needed to better understand whether neuroimmune mechanisms underly the pathophysiology of nicotine addiction-related behaviors.

### Alcohol

Similar to cocaine and methamphetamine, alcohol (ethanol) promotes inflammation and immune system dysfunction. Alcohol and its metabolites induce oxidative stress, increase systemic endotoxin levels, and promote the release of inflammatory peptides. Together, these effects contribute to liver disease, bone and muscle disease, cardiovascular disease, reproductive disorders, and neuroinflammation (González-Reimers et al., 2014). Many studies have revealed alcohol-induced changes in immunoregulatory miRNAs, cytokines, and other signaling pathways associated with neuroinflammation, and increasing evidence suggests that alcohol-induced neuroinflammation contributes to brain damage and neurodegeneration that is observed in individuals with severe alcohol use disorders (AUDs) (Vallés et al., 2006; Tajuddin et al., 2014; Orio et al., 2019).

NF-κB regulates alcohol-induced changes in immune-related gene expression, either through direct interactions with NF-κB-specific binding sites on many immune-related genes (e.g., IL-1β, TNFα, and MCP1) or with miRNAs to indirectly regulate immune-related gene expression. For example, the miRNA miR-155 is induced by NF-κB activation and plays a role in alcohol-induced dysregulation of TNFα and MCP1 expression. Specifically, chronic alcohol intake increases TNFα and MCP1 expression within the cerebellum, an effect mediated by alcohol-induced activation of miR-155 (Lippai et al., 2013). This effect was further validated in this study using miR-155 knockout mice, where alcohol-induced TNFα and MCP1 elevations were prevented in these animals following alcohol treatment. Also known for its role in alcohol-induced liver diseases (for review see Hartmann and Tacke, 2016), miR-155 has been shown to promote cytokine release through TLR4 activation (Lippai et al., 2013). TLR4, which is a potent mediator of alcohol-induced neuroinflammation (Alfonso-Loeches et al., 2010), participates in alcohol-induced, long-term synaptic remodeling during adolescence (Montesinos et al., 2016), promotes leukocyte infiltration across the blood–brain barrier (BBB) in the presence of alcohol (Alfonso-Loeches et al., 2016), and participates in alcohol-induced autophagy and synaptic dysfunction during development (Montesinos et al., 2018). Furthermore, alcohol increases the release of TLR4 and cytokines from astrocytes by mediating the release of astrocyte-derived extracellular vesicles (EVs) containing these inflammation-related proteins (Ibáñez et al., 2019). Such mechanisms are capable of altering the physiological state of neurons and has the ability to enhance alcohol-induced neuroinflammation. Interestingly, alcohol does not induce EV release in TLR4-deficient astrocytes, further implicating TLR4 as an important component of alcohol-induced neuroinflammation (Ibáñez et al., 2019). Additionally, genetic elimination of TLR4 prevents damage to myelin and synapses within the PFC as well as neuroinflammatory processes induced by intermittent alcohol treatment (Montesinos et al., 2015), and inhibition of TLR4 using the opioid antagonist nalmefene has been shown to prevent alcohol-induced neuroinflammation (Montesinos et al., 2017). Taken together, TLR4 plays an essential role in mediating alcohol-induced neuroinflammation and may serve as a promising target for treatment in patients with AUDs.

Similar to other drugs of abuse, alcohol consumption increases LPS-induced inflammation, which has known interactions with TLR4 located within the hepatic system as well as on microglia and astrocytes (Monnig, 2017). Interestingly, while alcohol increases proinflammatory cytokine release within the mouse hippocampus, including TNFα, IL-1β and MCP-1, co-treatment with LPS (alcohol + LPS) further increases MCP-1 and IL-1β (Qin et al., 2008). Additionally, alcohol + LPS significantly decreases the number of cells within the dentate gyrus relative to controls, an effect not observed with alcohol or LPS treatment alone (Qin et al., 2008). The ionized calcium binding adapter molecule 1 (Iba-1) protein is expressed within microglia and is elevated in post-mortem brains of patients with AUD (He and Crews, 2008). Iba-1 expression is increased in a TLR4-dependent manner in response to ethanol, where 35 days of chronic intermittent ethanol (CIE) exposure enhanced Iba-1 expression within the PFC (Sanchez-Alavez et al., 2019). Conversely, a recent study showed that 15 days of CIE exposure plus 10 h of withdrawal decreases Iba-1 immunoreactivity within the prelimbic cortex (PrL), whereas no changes are observed in the NAc. This study also demonstrated that LPS exposure produces an enlargement of soma volume in Iba-1-expressing microglia within the PrL and NAc and a decrease in microglial density within the NAc (Siemsen et al., 2020). Taken together, these studies suggest that chronic alcohol exposure may prime both central and peripheral immune mechanisms to exhibit exacerbated immune responses to inflammatory insults such as LPS. As well, these studies suggest that alcohol exposure produces a unique profile of neuroimmune effects that are distinct from classic proinflammatory stimuli such as LPS and that the duration of alcohol exposure and withdrawal are important factors when considering alcohol-induced changes in neuroimmune function.

In addition to processes described above, alcohol activates nicotinamide adenine dinucleotide phosphate (NADHP) oxidase, an enzyme known to produce reactive oxygen species, thus contributing to oxidative stress, alcohol-induced cell damage, and neuroinflammation (Qin and Crews, 2012). As a common activator of glial cells within the brain, oxidative stress is promoted by inducible nitric oxide synthase (iNOS), a gene activated by pro-inflammatory cytokines induced by alcohol (Sun and Sun, 2001). Further, iNOS catalyzes the oxidation of alcohol into acetaldehyde and α-hydroxyethyl radicals (Porasuphatana et al., 2006), and acetyladehyde production promotes reactive oxygen species (ROS) formation that contributes to unbalanced cellular oxidative stress (Hernández et al., 2016; Yan et al., 2016). Through its activation of p38 mitogen-activated protein kinase (MAPK) and inhibition of extracellular signal-regulated kinases, acetaldehyde induces cytotoxicity by promoting apoptotic signaling and inhibiting cell survival pathways (Yan et al., 2016). Interestingly, inhibition of the phosphoinositide 3-kinase/protein kinase B (PI3K/Akt) pathway with glycine inhibits ROS production in young rats and reduces alcohol-induced neuroinflammation (Amin et al., 2016). Altogether, these studies highlight the complexities of ROS and oxidative stress induced by alcohol, and more studies investigating the role of these processes in alcohol addiction-related behaviors are warranted.

Preclinical models of alcohol consumption are largely reliant on three forms of self-administration paradigms, which include drinking-in-the-dark (DID), two-bottle choice (2BC), and operant conditioning for oral alcohol consumption. Each paradigm allows rodents to self-administer alcohol-containing solutions and have varying degrees of translational value. Pharmacological and genetic manipulations of neuroinflammatory targets have yielded promising results in reducing alcohol consumption across these different paradigms. For example, inhibition of microglia activation reduces alcohol consumption during the DID task in C57/BL6 male mice (Lainiola and Linden, 2017) and prevents relapse-like drinking in the 2BC task in male rats (Gajbhiye et al., 2018). Further, inhibition of microglial P2X7 receptor signaling also reduces alcohol consumption in C57/BL6 male mice during the DID paradigm (Lainiola and Linden, 2017). Dual knockout of IL-1 and TNFα receptors reduces social stress-induced increases in alcohol consumption during the 2BC task in male mice (Karlsson et al., 2017), and IL-6 knockout mice also show similar reductions (Blednov et al., 2012). Likewise, antagonism of the IL-1 receptor within the basolateral amygdala reduces alcohol consumption during DID in male C57/BL6 mice (Marshall et al., 2016). Similar reductions in alcohol consumption have also been observed using peroxisome proliferator activated receptor (PPAR) agonists, which generally promote anti-inflammatory responses. Specifically, PPARα activation with fenofibrate decreases alcohol consumption during the 2BC and DID tasks in both UChB male rats and C57/BL6 male mice (Karahanian et al., 2014; Blednov et al., 2016). PPARα activation with gemfibrozil also decreases 2BC alcohol consumption in male Sprague-Dawley rats (Barson et al., 2009). Lastly, treatment with ceftriaxone [a beta-lactam antibiotic with immunomodulatory mechanisms (Wei et al., 2012; Kaur and Prakash, 2017; Ochoa-Aguilar et al., 2018)] or the antioxidant *N*-acetylcysteine reduces relapse-like drinking in alcohol preferring P rats (Qrunfleh et al., 2013; Alhaddad et al., 2014) as well as alcohol seeking and alcohol-reinforced responding in Long-Evans rats (Lebourgeois et al., 2018). Altogether, these findings provide a critical foundation for future investigations into anti-inflammatory compounds that may reduce alcohol motivation and consumption. However, most of the studies discussed here were conducted in male subjects only, making it difficult to determine whether these effects would also be observed in females. Thus, further characterization of potential sex differences in both alcohol-induced neuroimmune responses as well as the effects of pharmacological and genetic manipulations on these responses are warranted.

While much preclinical evidence exists demonstrating alcohol-induced neuroinflammation and cellular toxicity as well as the effects of anti-inflammatory agents in reducing alcohol consumption, studies examining pharmacotherapies for treating alcohol-induced neuroinflammation in humans are lacking. *N*-acetylcysteine (NAC) prevents alcohol-induced neuroinflammation in rats (Schneider et al., 2017) and human studies suggest that NAC may be useful for treating AUDs. Preclinically, NAC prevents alcohol withdrawal-induced increases in TNFα, IL-1β, IL-6, and IL-18 within the hippocampus and medial prefrontal cortex of rats (Schneider et al., 2017). As well, co-treatment with aspirin and NAC restores alcohol-induced impairments in glial glutamate transporter expression within the prefrontal cortex and prevents microglial activation (as measured through morphological alterations) within the hippocampus of rats (Israel et al., 2019). Parallel to these preclinical findings, NAC has also shown efficacy within humans with AUDs for reducing alcohol intake and withdrawal symptoms (Back et al., 2016; Squeglia et al., 2016, 2018); however, studies have not examined whether NAC reduces inflammation in humans. NAC’s poor bioavailability is a significant clinical limitation that may impair its efficacy. Thus, improving its bioavailability with novel delivery vectors such as nanoparticles may enhance its anti-inflammatory properties and, consequently, its therapeutic efficacy (Markoutsa and Xu, 2017). Similar to NAC, the lipid transmitter oleoylethanolamide (OEA), which is primarily generated by phospholipid cleavage, reduces alcohol-mediated neuroinflammation. By modulating oxidative stress, neuroinflammation, glial cell activation, as well as neurotransmission, OEA has neuroprotective properties, such as blocking alcohol-induced upregulation of TLR4 and subsequent proinflammatory signaling (Orio et al., 2019; Orio, 2020). OEA provides neuroprotection of the frontal cortex in alcohol-exposed rats by inhibiting NF-κB and TLR4 signaling pathways (Antón et al., 2017). Interestingly, systemic OEA treatment reduces operant responding for alcohol and prevents alcohol-induced withdrawal symptoms in rodents (Bilbao et al., 2016).

Another novel treatment target that is being investigated both clinically and preclinically for its use in treating AUDs is phosphodiesterases (PDEs). PDEs are upstream modulators of intracellular cyclic nucleotides, such as cyclic AMP (cAMP) and cyclic GMP (cGMP), as well as immunomodulators that underly alcohol reward and toxicity (Wen et al., 2018). Specifically, PDEs catalyze the hydrolysis of cAMP and cGMP, decreasing their intracellular levels and thus downstream signal transduction. As well, PDEs (e.g., PDE4) are involved in ethanol-mediated inflammatory responses (Gobejishvili et al., 2008; Avila et al., 2017). Evidence suggests that ethanol exposure alters cAMP signal transduction by modulating adenylate cyclase (Yang et al., 1996; Asher et al., 2002; Asyyed et al., 2006). This modulatory effect may depend on the amount of ethanol exposure as acute ethanol promotes cAMP signal transduction whereas chronic ethanol attenuates this signaling (Saito et al., 1987; Yang et al., 1996, 1998; Pandey, 2004). Preclinical studies show that reductions in cAMP and cGMP levels, caused by hyperactivity of PDEs, promote excessive ethanol use. Given their link to alcohol seeking, several studies have attempted to pharmacologically identify which PDEs modulate alcohol consumption (Hu et al., 2011; Wen et al., 2012; Blednov et al., 2014; Bell et al., 2015; Logrip, 2015). Other studies have attempted to describe genetic differences in PDEs between alcohol-preferring and non-preferring mouse lines (Mulligan et al., 2006). Importantly, pharmacological inhibition of PDE4 (Hu et al., 2011; Wen et al., 2012; Blednov et al., 2014) and PDE10 (Bell et al., 2015; Logrip, 2015) reduce ethanol intake in rodents. Given the results of these preclinical findings, two PDE inhibitors are undergoing clinical trials for their therapeutic efficacy in treating AUDs. Specifically, the PDE inhibitor Ibudilast, a non-specific PDE inhibitor that targets PDE3, PDE4, PDE10, and PDE11, is currently within Phase I (ClinicalTrials.gov, 2021a), while the PDE4 inhibitor Apremilast is currently within Phase II (ClinicalTrials.gov, 2021b). For additional information regarding PDEs and alcohol, see the in-depth review by Wen et al. (2018). Taken together, targeting neuroinflammation and reducing oxidative stress may be a promising therapeutic strategy for individuals with AUDs by reducing the underlying effects of alcohol on neuroinflammation.

### Opioids

Opioids are a class of drugs that possess a very high abuse liability and have been at the center of an ongoing public health crisis. Between the years 2000 and 2017, yearly opioid overdose-related deaths rose to more than 47,000 within the United States, and 21–29 percent of individuals who are prescribed opioids to manage chronic pain misuse them (Vowles et al., 2015; National Institute on Drug Abuse [NIDA], 2020c). In response to this crisis, considerable preclinical research has revealed many neuronal and non-neuronal mechanisms underlying opioid reward and reinforcement, including neuroimmune mechanisms. This research has advanced our understanding of the pathophysiology of opioid use disorders (OUDs) as well as other comorbid chronic pain and inflammatory conditions. For example, the opioid receptor antagonist naltrexone, which has been used as a medication to treat both OUDs and AUDs, attenuates inflammation and pain associated with chronic autoimmune disorders and cancer (Li et al., 2018; Patten et al., 2018). Several groups have recently provided thorough, in-depth reviews of the many neuroimmune mechanisms involved in OUDs (Plein and Rittner, 2018; Eidson and Murphy, 2019; Hofford et al., 2019; Zhang et al., 2020). Thus, for the purposes of this review, we will summarize key findings within the last 5 years, and emphasize recent advancements within the last 2 years where possible, to highlight current trends in the field and offer our perspectives on future directions regarding novel neuroimmune mechanisms of OUDs.

TLR4 is known to play a critical role in the rewarding and reinforcing effects of opioids, and TLR4 antagonists may be efficacious pharmacotherapies to treat OUDs (Hutchinson et al., 2012; Wang et al., 2012; Bachtell et al., 2015). Specifically, a study by Hutchinson et al. (2012) showed that inhibition of TLR4 and its downstream myeloid differentiation primary response 88 (MyD88)-dependent signaling suppresses opioid-conditioned place preference and self-administration as well as morphine-induced increases in NAc extracellular dopamine. This study was paralleled by another study demonstrating that morphine induces neuroinflammation through activation of TLR4 signaling and that inhibition of TLR4 signaling can enhance the analgesic properties of morphine (Wang et al., 2012). TLR4 is also involved in opioid reinforcement, tolerance, and withdrawal. For example, knockdown of TLR4 within the ventrolateral periaqueductal gray (vlPAG) increases glutamic acid decarboxylase mRNA within the PAG and decreases withdrawal symptoms in morphine-exposed rats (Liu Q.F. et al., 2016). As well, chronic morphine exposure stimulates TLR4 and recruits TNFα signaling within the PAG to enhance neuroinflammation, downregulate glutamate transporters, and increase morphine tolerance (Eidson et al., 2017). Within the VTA, TLR4 signaling also facilitates the acquisition and maintenance opioid CPP, which may depend on downstream signal transducer and activator of transcription 3 (STAT3) signaling (Chen et al., 2017). Interestingly, genetic deletion of Myd88, in microglia of mice impairs extinction learning and enhances reinstatement of morphine CPP (Rivera et al., 2019). These findings are seemingly conflicting with those of Hutchinson et al. (2012) as highlighted above which used a Myd88 knockout mouse model that lacked cell-type specificity. Nevertheless, it is likely that neuroimmune influences over addiction-related behaviors, such as those mediated by TLR4-Myd88 signaling, are cell type-specific and brain region-dependent. Moreover, there are likely sex-specific neuroimmune mechanisms that may influence opioid addiction-related behaviors and opioid treatment outcomes for chronic pain (Posillico et al., 2015; Doyle et al., 2017). While there is still some conflicting evidence regarding the efficacy of TLR4 antagonists for reducing opioid craving and relapse (Yue et al., 2020), future research should explore the efficacy of TLR4 antagonists as medications that supplement other treatment approaches for OUDs and comorbid chronic pain.

Several recent investigations have identified many cytokines and other immune-related signaling mechanisms that may regulate opioid reinforcement and motivation. For example, a recent study using a rodent model of fentanyl self-administration showed that the NAc and HPC are particularly vulnerable to changes in the expression of a number of immune markers, such as increased IL-1β, TNFα, and IFN-γ within the NAc and decreased GM-CSF and IFN-γ in the HPC (Ezeomah et al., 2020). While this study did not directly manipulate any of these neuroimmune substrates, expression of IL-1β and IL-6 within the NAc, as well as IFN-β and various interleukins within the HPC, correlate positively and negatively (respectively) with fentanyl intake. Similarly, another study using single-cell qRT-PCR analyses demonstrated that morphine withdrawal is associated with increased expression of neuroinflammatory genes, such as *Tnf*, as well as increased TNFα protein expression within neurons, microglia, and astrocytes from the central amygdala. Among these cell types, astrocytes exhibited the largest changes in neuroimmune gene expression (O’Sullivan et al., 2019). Such findings implicate opioid-induced neuroinflammatory interactions with neural reward systems that are known to drive drug-seeking behavior (Koob and Volkow, 2016). Rodent self-administration models with the prescription opioid oxycodone have shown upregulation within both the dorsal and ventral striatum of numerous immune-related genes using RNAseq and qPCR techniques [e.g., *Ccr5*, *Icam1*, *Cybb* (Zhang Y. et al., 2017)], as well as changes within the NAc and CPu in the expression of various integrin, semaphorin, and ephrin genes that play a role in neurodevelopment, cell migration, structural regulation of synapses, and immune signaling (Yuferov et al., 2018).

Several studies examining the efficacy of various immunomodulatory treatments have also provided evidence for immune signaling as an important mechanism underlying opioid addiction-related behaviors. For example, *N*-acetylcysteine effectively inhibits heroin-seeking behavior akin to other drugs of abuse (Zhou and Kalivas, 2008; Hodebourg et al., 2019). Similarly, IL-10 overexpression within the NAc reduces remifentanil self-administration in rats (Lacagnina et al., 2017) and intra-NAc treatment with the antibiotic and microglia inhibitor minocycline inhibits drug-primed reinstatement of morphine CPP in rats (Arezoomandan and Haghparast, 2016). Clinical studies also support the use of anti-inflammatory medications to treat OUDs. For instance, the anti-inflammatory phosphodiesterase inhibitor ibudilast reduces heroin cravings and self-reported pain ratings among opioid-dependent individuals (Metz et al., 2017). Another study suggests beneficial effects of ibudilast on withdrawal symptoms in opioid-dependent individuals undergoing detoxification (Cooper et al., 2016). The cyclooxygenase-2 (COX-2) inhibitor celecoxib may also reduce opioid cravings among individuals undergoing detoxification (Jafari et al., 2017). Overall, the studies described here provide evidence supporting the conclusion that neuroimmune mechanisms may be involved in the pathophysiology of OUDs, particularly regarding tolerance to both the rewarding and analgesic properties of opioids, as well as withdrawal from chronic opioid use. Nevertheless, there is still a significant gap in the literature regarding the efficacy of novel immunomodulatory medications, and particularly anti-inflammatory agents, in reducing opioid craving and relapse as well as tolerance to the analgesic effects of opioids. Specifically, the changes in neuroimmune function in acute versus protracted abstinence from chronic opioid self-administration and the functional role of such changes on opioid craving and relapse are not well understood. As well, sex differences within these processes are poorly understood. Future studies are needed to improve our understanding of how neuroimmune mechanisms may contribute to opioid relapse.

## Neuroimmune Mechanisms and Substance Use Disorder Comorbidities

Many individuals who are diagnosed with a SUD often suffer from other physical or psychiatric comorbidities that can hinder successful treatment outcomes. According to the United States Substance Abuse and Mental Health Services Administration (SAMHSA), over 9.2 million American adults had both a SUD and a comorbid mental illness such as anxiety or depression as of 2018 (Substance Abuse and Mental Health Services Administration, 2019). Furthermore, physical comorbidities such as chronic pain and HIV are more difficult to treat among those with SUDs (National Institute on Drug Abuse [NIDA], 2020a). Convergent findings from numerous clinical and preclinical studies have implicated immune dysfunction as a possible shared mechanism linking these comorbidities to the pathophysiology of SUDs. Here, we briefly highlight recent studies demonstrating immune system dysfunction in disorders that are commonly comorbid with SUDs, focusing primarily on major depressive disorder (MDD), posttraumatic stress disorder (PTSD), chronic pain, and HIV. The studies discussed here may provide useful insights into commonly shared mechanisms that facilitate the pathogenesis of these comorbidities as well as increase SUD vulnerability.

### Depression

The idea that depression (also referred to as Major Depressive Disorder, or MDD) may be influenced by immune system dysfunction dates back to the early 1990’s, when Ronald Smith published his seminal paper on the “macrophage theory of depression” (Smith, 1991). Among the evidence described by Smith included the observations that experimental volunteers who were administered monocyte/macrophage-derived cytokines developed many of the symptoms of MDD and that individuals with chronic inflammatory diseases such as rheumatoid arthritis are more likely to have depression. Ahead of his time, Smith also postulated that the “food-gut-allergy” axis may be involved in the pathogenesis of MDD. While outside the scope of this review, recent studies have elucidated key mechanisms of the gut microbiome in human health and disease, including its role in the etiology of psychiatric illnesses (Singh et al., 2017; Cryan and de Wit, 2019). Both systemic inflammation induced by xenobiotic substances and inflammatory disease states, such as in multiple sclerosis and spinal cord injury, are associated with depressive symptoms in humans (Allison and Ditor, 2015; Engler et al., 2017; Morris et al., 2018). Other inflammatory conditions such as irritable bowel syndrome and psoriasis are associated with higher prevalence rates of depression, anxiety, and substance abuse, suggesting that inflammatory mechanisms may reside at the intersection between these disorders (Hayes and Koo, 2010; Fond et al., 2014; Knight et al., 2015). MDD is often comorbid with AUDs in particular, and many studies implicate neuroimmune dysfunction as a significant pathophysiological feature of this comorbidity (for review, see Neupane, 2016). Individuals with MDD also show elevated serum levels of various immune factors such as IL-6 (Engler et al., 2017; Ye et al., 2018) and TNFα (Fan et al., 2017; Zou et al., 2018), and IL-6 in particular has been implicated in depression across a number of clinical and preclinical studies (for review, see Hodes et al., 2016). A recent example is that stress-susceptible mice treated systemically with an IL-6 antibody show significant reductions in depressive-like behaviors in the chronic social defeat stress model of depression, which generally upregulates systemic levels of IL-6 (Hodes et al., 2014; Stewart et al., 2015; Zhang J.C. et al., 2017). As well, social stress and drugs of abuse can downregulate the expression of cell adhesion and tight junction proteins such as collagen-IV and claudin-5, leading to infiltration of immune factors like IL-6 from the periphery (Menard et al., 2017; Rodríguez-Arias et al., 2017). In a recent preclinical study, mice exposed to chronic unpredictable stress exhibited elevated levels of TNFα within the HPC and increased depressive- and anxiety-like behaviors, which were attenuated by minocycline treatment (Zhang et al., 2019). Interestingly, minocycline has also been shown to reduce both METH- and alcohol-seeking behavior in rodents (Fujita et al., 2012; Attarzadeh-Yazdi et al., 2014; Gajbhiye et al., 2017).

Serum levels of TNFα are also elevated in individuals with MDD (Tuglu et al., 2003), and elevated levels of TNFα may be associated with treatment-resistance, particularly with selective serotonin reuptake inhibitors (SSRIs) (Tuglu et al., 2003; O’Brien et al., 2007; Eller et al., 2008). Interestingly, TNFα levels are positively correlated with midbrain serotonin transporter (SERT) availability in humans (Krishnadas et al., 2016), suggesting a possible immunomodulatory mechanism that underlies dysregulated serotonin neurotransmission in depression. The SSRIs fluoxetine and escitalopram inhibit M1 proinflammatory activation and promote M2 anti-inflammatory activation of microglia *in vitro* (Su et al., 2015). As well, rats experiencing chronic mild stress exhibit elevated plasma levels of proinflammatory cytokines (IL-1β, IL-17, and TNFα), which are prevented by chronic fluoxetine treatment (Lu et al., 2017). This is mirrored by a recent meta-analysis demonstrating that patients with MDD who respond to antidepressant treatment show significantly decreased peripheral TNFα levels (Liu et al., 2020).

The studies above highlight that antidepressant SERT inhibitors exert immunomodulatory effects, and these effects may be related to their therapeutic efficacy. As well, several recent preclinical studies suggest that changes in SERT expression and/or activity alters glutamatergic plasticity and addiction-related behaviors. For example, SERT knockout (SERT^–/–^) rats, which exhibit depression- and anxiety-like behaviors (Kalueff et al., 2010), show decreased mRNA expression of key glutamatergic substrates within the habenula, including GLT-1, NMDA subunits (GluN1, GluN2A, and GluN2B), and AMPA subunits (GluA1 and GluA2) (Caffino et al., 2019). Moreover, SERT^–/–^ rats exhibit greater cocaine self-administration, and cocaine self-administering SERT^+/+^ rats showed reduced mRNA levels of genes encoding for GLT-1 and GluN1 to the levels of SERT^–/–^ rats (Caffino et al., 2019). Another recent study utilizing the bilateral olfactory bulbectomy (OBX) model of depression in combination with cocaine self-administration showed that treatment with escitalopram dose-dependently reduces cue-induced cocaine-seeking behavior in both OBX and sham control rats as well as burst responding on day 1 of extinction training (Jastrzêbska et al., 2017). As illustrated in Figure 1, glutamatergic signaling interfaces with neuroimmune function in glial cells, and serotonergic dysfunction in MDD (e.g., altered SERT function) could lead to dysregulated glutamatergic signaling and, subsequently, altered neuroimmune function. Nevertheless, this remains largely speculative, and more studies are needed to fully investigate whether serotonergic substrates directly or indirectly alter neuroimmune function within animal models of MDD and SUDs and if such neuroimmune mechanisms are relevant to the pathophysiology of this comorbidity.

Although it is unclear whether specific cytokine inhibitors alone would be broadly effective treatments for MDD (Raison et al., 2013; Smolen et al., 2014), these studies support the notion that immunopharmacology may be an efficacious adjunctive treatment strategy in combination with existing anti-depressants for treating co-morbid MDD and SUDs. Ketamine, which has demonstrated efficacy in cases of chronic, treatment-resistant depression, exhibits a broad spectrum of anti-inflammatory effects (e.g., TNFα and IL-6 inhibition) in both clinical and preclinical studies (Murrough et al., 2013; Tan et al., 2017; Chen et al., 2018). Indeed, broad-spectrum immunomodulators, as opposed to cytokine-specific antibodies for example, may prove to be effective treatment strategies for MDD. For instance, drugs that confer a wide range of antioxidant and anti-inflammatory effects, such as the cysteine prodrug *N*-acetylcysteine, show clinical promise as adjunctive treatments for MDD, bipolar depression, and for SUDs (Berk et al., 2011, 2014; Magalhães et al., 2011; Carvalho et al., 2013; Tomko et al., 2018). Nevertheless, more studies are needed to further examine whether such treatment strategies are effective in cases of comorbid MDD and SUDs.

### Posttraumatic Stress Disorder

Akin to MDD, posttraumatic stress disorder (PTSD) is a comorbidity experienced by many individuals with SUDs. PTSD is associated with systemic elevation of inflammatory markers that may underly the pathophysiology of the disorder (for review, see Hori and Kim, 2019). A recent meta-analysis by Passos et al. (2015) revealed that serum IL-6, IL-1β, and interferon gamma (IFN-γ) are all elevated in individuals with PTSD compared to healthy controls. IL-6 is also positively correlated with disease severity and IL-1β with disease duration. Furthermore, when MDD comorbidity is controlled for, individuals with PTSD show elevated TNFα, IL-6, and IL-1β (Passos et al., 2015). Genetic studies in humans also provide converging evidence in support of immune dysregulation in PTSD. For example, a recent genome-wide association study identified significant gene loci (e.g., ANKRD55) associated with immune disorders such as rheumatoid arthritis and psoriasis in individuals with PTSD (Stein et al., 2016).

Preclinical studies also implicate both central and peripheral immune activation in the pathophysiology of PTSD. For instance, fear memory retrieval in mice is associated with increased circulating IL-6 and inhibition of IL-6 signaling can improve extinction learning (Young et al., 2018). In addition, many inflammatory and innate immune pathways such as interferons, interleukins, and TNFα remain activated long after social stress exposure in mice, while anti-inflammatory mediators such as TGFβ are inhibited (Muhie et al., 2017). Interestingly, predator stress exposure in mice induces a state of immunosuppression within the brain, but acute “priming” of the immune system with LPS prior to this form of stress exposure has no effect on later avoidance behavior (Deslauriers et al., 2017). This suggests that chronic stress experienced with PTSD, as opposed to acute trauma exposure itself, may underly the persistence of immune dysregulation seen in individuals with PTSD.

Other studies utilizing predator-based psychosocial stress models also highlight the significance of prior drug experience in modulating the effects of stress on drug taking later in life. For example, a recent study demonstrated that rats exposed to chronic predator stress showed enhanced ethanol preference (over sucrose) compared to unstressed controls only when they had ethanol experience with two-bottle choice *prior* to stress exposure (Zoladz et al., 2018). A question that remains is whether prior stress and drug experience interact to dysregulate immune signaling in a way that promotes stress susceptibility and/or subsequent increases in addiction-related behaviors. One recent study sheds some light on this topic, demonstrating that early life stress in the form of maternal separation increases PFC and NAc TNFα levels in males, but not females, and enhances cocaine CPP in maternally stressed males. As well, this study showed that systemic inhibition of TNFα restores cocaine CPP to control levels (Ganguly et al., 2019). This supports another recent study showing that early life social stress in mice can sensitize both central and peripheral immune responses to cocaine within the VTA and that humans with CUDs that experienced childhood social stress show elevated expression of inflammatory markers (e.g., TLR4, TNFα, IL-1β, and IL-6) (Lo Iacono et al., 2018). Indeed, these findings highlight that a history of chronic stress or trauma, especially during adolescence, may cause aberrant changes in immune function that could increase SUD susceptibility later in life.

As highlighted throughout this review and illustrated in Figure 1, glutamate dysfunction is a consistent feature observed across drugs of abuse and recent evidence implicates metabotropic glutamate receptor 5 (mGlu5) in the pathophysiology of PTSD and neuroimmune function. Indeed, dysfunction of glutamatergic plasticity is also thought to be involved in pathophysiology of PTSD (for review, see Averill et al., 2017). Microglia, astrocytes, and peripheral immune cells express mGlu5, which promotes anti-inflammatory responses (Fazio et al., 2018). Interestingly, a recent study showed that neuroinflammation induced by traumatic brain injury in mice is reduced by mGlu5 PAM treatment and that the mechanism of action involves Akt/GSK-3β/CREB signaling (Bhat et al., 2021). This corroborates previous evidence showing that stimulation of mGlu5 reduces LPS-induced microglial activation and proinflammatory signaling (Byrnes et al., 2009). A recent study by Schwendt et al. (2018) showed that rats resilient to stress induced by predator odor exposure exhibit increased *mGlu5* gene expression within the amygdala and mPFC. As well, stress-susceptible animals show enhanced cue-induced reinstatement of cocaine seeking that is not attenuated by the β-lactam antibiotic ceftriaxone, which is a drug known to reliably suppress this behavior (Knackstedt et al., 2010; LaCrosse et al., 2016; Schwendt et al., 2018). Importantly, treating stress-susceptible animals with a mGlu5 positive allosteric modulator (PAM) along with ceftriaxone and fear extinction successfully prevented cue-induced reinstatement of cocaine seeking (Schwendt et al., 2018). Another recent study parallels these findings, showing that early life stress in rats causes these animals to spend less time in the light area of a light-dark box and to exhibit lower *mGlu5* gene expression within the amygdala. This study also showed that time spent in the light area is positively correlated with *mGlu5* gene expression within the amygdala (Buonaguro et al., 2020). One question that remains is whether such mGlu5-mediated neuroimmune mechanisms are therapeutic in cases of comorbid PTSD and SUDs. Given the putative role of glutamatergic substrates such as mGlu5 in both PTSD and SUDs, further investigation into the neuroimmune mechanisms of this receptor system within animal models of comorbid PTSD and SUDs is warranted.

Novel drugs that modulate immune signaling have shown stress-reducing effects in preclinical models. For example, a recent study showed that intranasal oxytocin treatment following single prolonged stress (SPS) exposure is sufficient to reverse physiological and behavioral deficits such as increased central and peripheral proinflammatory cytokine expression, reduced plasma corticosterone levels, and impaired extinction learning (Wang S.C. et al., 2018). Additionally, SPS in rats treated with a cyclooxygenase-2 (COX-2) inhibitor exhibit decreased apoptosis in the HPC, reduced levels of proinflammatory cytokines in the HPC, and decreased anxiety-like behavior (Wang M. et al., 2018). Other preclinical studies have also shown similar effects on anxiety-like behavior and proinflammatory cytokine expression within the brain using COX-2 inhibitors (Gamble-George et al., 2016; Lee et al., 2016), and human studies suggest that anti-inflammatory treatments such as COX-2 inhibitors may be effective in reducing depression symptoms (Köhler et al., 2014). Interestingly, the antioxidant *N*-acetylcysteine (NAC) was recently shown to inhibit conditioned stress-induced cocaine and alcohol seeking when administered during or immediately following restraint stress (Garcia-Keller et al., 2020), which raises the question as to whether suppression of oxidative stress and inflammation via NAC treatment is therapeutic in this context. Importantly, NAC is currently being investigated clinically for its use in treating comorbid AUDs and PTSD (Back et al., 2020). Drugs with immunomodulatory activity, such as monoclonal antibodies, glucocorticoids, and cannabinoids, are also being investigated for their use in the treatment of PTSD (Hori and Kim, 2019). Overall, these studies provide a firm rationale for future investigations into immunomodulatory medications to treat symptoms of PTSD. However, it remains largely unknown whether such treatment strategies are efficacious in treating cases of comorbid PTSD and SUDs. Indeed, use of psychotropic substances can explain a significant degree of heterogeneity across studies examining proinflammatory cytokine expression in individuals with PTSD (Passos et al., 2015). Thus, future investigations should consider the neuroimmune intersections of comorbid PTSD and SUDs that may impact the therapeutic efficacy of treatments.

### Chronic Pain

Substance abuse and misuse, particularly with opioids, is more common among individuals with chronic pain. For example, an estimated 10% of individuals living with chronic pain also misuse prescription opioids, and chronic pain interacts with mesocorticolimbic reward circuits and stress pathways to increase SUD vulnerability (for review, see Elman and Borsook, 2016; Garland et al., 2013). Chronic pain, defined as pain lasting longer than 3 months or beyond the period of normal tissue healing, affects as much as 43% of adults within the United States (Dowell et al., 2016). While the prescribing of opioids has declined since 2012 (Zhu et al., 2019), these medications are still commonly used to treat chronic pain and opioid-related overdoses and deaths still remain high (Scholl et al., 2018). Inflammation and immune system dysfunction are defining features of many forms of chronic pain (Marchand et al., 2005; Totsch and Sorge, 2017) and several recent studies have identified immunomodulatory pain mechanisms that may extend our understanding of the pathophysiology of SUDs (particularly OUDs). For example, a recent preclinical study using the complete Freund’s adjuvant (CFA) model of inflammation found that rats experiencing chronic inflammation require higher doses of heroin (150 μg/kg) to achieve significant increases in NAc core dopamine release. As well, this study showed that inflammatory pain impairs MOR-mediated inhibition of GABA transmission within the VTA and enhances heroin self-administration at higher doses without impacting sucrose self-administration (Hipólito et al., 2015). This is consistent with another study demonstrating that chronic neuropathic pain decreases MOR1 availability and expression within the rat CPu and motor cortex, which correlates positively with sucrose preference in nerve-injured animals (Thompson et al., 2018). Converging evidence suggests that chronic inflammatory pain may also impair the analgesic effects of opioids (Steele et al., 2002; DeLeo et al., 2004; Zhang et al., 2004). Studies also show that the MOR antagonist naltrexone may reduce chronic inflammatory pain through inhibition of T and B cell proliferation and TLR4 signaling (Tawfik et al., 2016; Patten et al., 2018), suggesting a potentially promising role for adjunctive immunomodulatory therapies in chronic pain management. Taken together, inflammatory mechanisms can lead to dysregulations in endogenous opioid receptor signaling that can facilitate chronic pain and lead to escalated drug intake over time and, subsequently, increased risk for drug dependence and substance abuse. Thus, use of anti-inflammatory medications as adjunctive therapies to improve chronic pain management may have the added benefit of reducing the risk of addiction to opioid pain medications.

Chronic pain and antinociceptive opioid drugs are subject to important sex differences that may have underlying neuroimmune mechanisms. Indeed, some of the most common chronic pain conditions such as migraine, low back pain, and neck pain, are vastly overexpressed in women (Mogil, 2012). In fact, women are overall more likely to use prescription opioids than men (Serdarevic et al., 2017) and women may also experience greater levels of pain than men in a manner that may be dependent on opioid withdrawal (Huhn et al., 2019). Preclinical studies have identified sexual dimorphism of cellular responses to opioids that may explain some of the sex differences in pain observed in humans. For example, a recent study demonstrated that female rats exhibit increased basal and LPS-induced microglial activation within the PAG, and that basal activation of microglia is a significant predictor of the ED_50_ of morphine’s antinociceptive effects (Doyle et al., 2017). Moreover, this study showed that naloxone inhibition of TLR4 within the PAG significantly improves the antinociceptive effects of morphine. T cells may also underly observed sex differences in opioid analgesia, with one recent study demonstrating that T-cell-deficient mice exhibit impaired opioid-induced analgesia that is more pronounced in females (Rosen et al., 2019). Other non-opioidergic, sex-specific neuroimmune mechanisms underlying chronic pain have been identified, which could have significant implications for clinical treatment of pain and SUDs in human populations. For instance, ATP signaling via P2X4 receptors on microglia has been shown to be essential for nerve injury-induced allodynia in males but not in females (Mapplebeck et al., 2018). However, human studies have shown that the serotonin and norepinephrine reuptake inhibitor duloxetine, which also inhibits P2X4 signaling (Yamashita et al., 2016), reduces pain in women with fibromyalgia (Arnold et al., 2007; Bidari et al., 2019). This highlights the complexity of sex differences in the neuroimmune regulation of pain and suggests that perhaps dynamic immune interactions with monoamine neurotransmitter systems play a significant modulatory role in chronic pain between males and females (Lei et al., 2011; Packiasabapathy and Sadhasivam, 2018).

Neuromodulatory systems such as the endocannabinoid system (ECS) have also garnered significant attention for their role in chronic pain. Conflicting clinical and preclinical studies exist regarding sex differences in the efficacy of cannabinoids as chronic pain treatments (for review, see Cooper and Craft, 2018). The efficacy of such treatments likely depend upon the route of administration, type of pain, and locus of pain (Romero-Sandoval et al., 2017). Nevertheless, the ECS is well regarded for its role in regulating neuroimmune signaling and pain. For example, cannabinoid receptors such as CB1 and CB2 are expressed in CNS regions involved in nociception (e.g., PAG, amygdala, dorsal root ganglion, thalamus, etc.) and these receptors exert primarily anti-inflammatory effects (Burstein and Zurier, 2009; Barrie and Manolios, 2017). Cannabis has been proposed as a potential adjunctive or alternative therapeutic for OUDs, particularly in cases of comorbid chronic pain (Wiese and Wilson-Poe, 2018), although this has been disputed (Olfson et al., 2018; Wilson et al., 2018; McBrien et al., 2019). Nevertheless, cannabidiol (CBD), which is a specific chemical constituent of cannabis that has no intoxicating psychoactive properties, exerts anti-inflammatory activity (Nichols and Kaplan, 2020) that has shown significant promise as a potential therapeutic for OUDs (Hurd et al., 2015). Altogether, these studies underscore the need for future investigations into the use of immunomodulatory therapeutics in the treatment of chronic pain, either alone or as adjunctive therapies. Importantly, these medications may be useful in the treatment of comorbid chronic pain and SUDs, where common immune mechanisms may underly the pathophysiology of both conditions.

### Human Immunodeficiency Virus (HIV)

HIV is another physical comorbidity that can significantly impede treatment outcomes for SUDs and vice versa. According to the United States Centers for Disease Control and Prevention (CDC), over 1 million people in the United States are currently living with HIV and 1 in 7 of those affected are unaware of their HIV status (Centers for Disease Control and Prevention, 2019). Globally, nearly 40 million people are currently living with HIV, and individuals who inject drugs are at a 22 times greater risk for acquiring HIV (UNAIDS, 2019). Importantly, HIV still remains a significant public health concern among minority and socioeconomically disadvantaged populations (National Institute on Drug Abuse [NIDA], 2012; Durvasula and Miller, 2014). As well, drug abuse (particularly psychostimulant abuse) is associated with higher rates of risky behaviors such as unsafe sexual practices that can increase HIV risk (Centers for Disease Control and Prevention, 2018).

Modern antiretroviral therapy (ART) has proven successful at suppressing viral load and mitigating the transition to acquired immunodeficiency syndrome (AIDS), allowing many individuals living with HIV to live relatively normal lives. However, ART does not readily cross the BBB, rendering the brain vulnerable to chronic HIV infection (Atluri et al., 2015). Specifically, drugs of abuse such as cocaine can increase BBB permeability (Zhang et al., 1998) and facilitate invasion (An and Scaravilli, 1997) and viral replication (Sahu et al., 2015) of HIV in the brain even during the early asymptomatic stages of HIV infection. This process can happen rapidly and lead to chronic CNS infection that results in neuroimmune activation and, in many cases, HIV-associated neurocognitive disorders (HAND) (Hong and Banks, 2015; Zayyad and Spudich, 2015). Cocaine facilitates HIV replication through a NF-κB-dependent mechanism (Fiume et al., 2012; Sahu et al., 2015) and impairs the innate immune response of astrocytes to infiltrating viruses such as HIV through increased oxidative stress when tested *in vitro* (Cisneros et al., 2018). Drugs of abuse also exacerbate the effects of the HIV proteins *trans*-activator of transcription (Tat) and gp120 in inducing oxidative stress, microgliosis, and astrogliosis (Aksenov et al., 2001, 2003; Shah et al., 2013; Samikkannu et al., 2015; Zeng et al., 2018). Moreover, HIV Tat primes and activates proinflammatory responses in microglia via the NLR family pyrin domain containing 3 (NLRP3) inflammasome (Chivero et al., 2017), and dopamine can activate NF-κB and prime the NLRP3 inflammasome in macrophages (Nolan et al., 2020) (however, see Zhu et al., 2018). This could be exacerbated by drugs of abuse that promote dopamine transmission within areas of the brain that are particularly vulnerable to HIV, such as the striatum (Nolan and Gaskill, 2019). Clinically, the mitochondrial protein translocator protein (TSPO), which has garnered interest as a biomarker of neuroinflammation in human imaging studies (Werry et al., 2019), is upregulated in people living with HIV (PLWH) and increased TSPO within the hippocampus, amygdala, and thalamus is associated with impaired cognition (Vera et al., 2016). Other studies suggest that impaired cognitive function is associated with enhanced TSPO binding in frontal and temporal regions of the brain in PLWH (Garveya et al., 2014; Rubin et al., 2018). It remains unclear whether combined HIV and active drug use, compared to drug abstinence, produces differential profiles of neuroinflammation and immune activation to confer increased vulnerability to HAND and drug relapse. Nevertheless, many preclinical studies suggest that HIV and its protein products may exacerbate drug reward and reinforcement as well as drug-induced neuroadaptations that may impede successful SUD treatment outcomes.

Growing evidence suggests that HIV and its protein products interact with brain reward circuitry to contribute to dysregulated reward-seeking behavior and cognition. For example, HIV-1 transgenic rats, which constitutively express HIV-1 viral proteins and model many HIV-associated neurological abnormalities observed in humans (Moran et al., 2014; Vigorito et al., 2015; McLaurin et al., 2018), exhibit increased dopamine affinity at striatal dopamine transporters and a leftward shift in the ascending limb of the cocaine dose response function, suggesting enhanced sensitivity to the reinforcing properties of cocaine (McIntosh et al., 2015). Another study using this transgenic rat model showed that these animals exhibit enhanced hyperexcitability of pyramidal neurons within the PFC following abstinence from cocaine (Wayman et al., 2016). As well, inducible Tat expression within the brain of transgenic mice is associated with enhanced METH-induced microglia activation as well as METH-induced locomotor sensitization (Kesby et al., 2017). Other studies using the inducible Tat mouse model have shown that induced Tat expression within the brain increases the magnitude of cocaine CPP and is sufficient to reinstate extinguished cocaine CPP (Paris et al., 2014a). Similar effects have been observed in females, albeit in an estrous cycle phase-dependent manner (Paris et al., 2014b). Similarly, inducible gp120 expression within the mouse brain is associated with the formation of METH conditioned place preference at lower METH doses compared to non-transgenic controls (Kesby et al., 2014). Many other preclinical studies have also demonstrated impaired cognitive function in animals exposed to HIV proteins and drugs of abuse, mimicking the cognitive dysfunction observed in humans with HAND (Moran et al., 2014; Kesby et al., 2015a, b, 2016; Paydary et al., 2016). Taken together, such studies suggest that HIV and its protein products may increase one’s sensitivity to the rewarding and reinforcing properties of psychostimulants and impair cognitive function.

In contrast to the studies above, a recent study using the HIV transgenic rat model suggests that HIV many promote a motivational state of apathy (Bertrand et al., 2018). However, one significant limitation to this study and studies like it is that the HIV transgenic rat model constitutively expresses HIV proteins throughout the CNS *prior* to the establishment of stable drug self-administration, which may produce profoundly different effects on mesocorticolimbic circuit function and neuroimmune signaling compared to the induction of viral protein expression within the CNS *after* drug self-administration is established or during a period of abstinence. Indeed, some individuals with preexisting SUDs contract HIV collaterally through drug use and risky behaviors associated with drug use (Centers for Disease Control and Prevention, 2018). Regardless, it is possible that HIV-induced perturbations to neuroimmune signaling described above may underly these neurobehavioral intersections between HIV and SUDs. Thus, immunopharmacology may be a promising avenue of future research into medications that inhibit the long-term neurocognitive deficits observed with chronic HIV infection as well as drug craving and relapse in cases of comorbid SUDs (Roederer et al., 1992; Ambrosius et al., 2019; Tripathi et al., 2020). Interestingly, ART itself possess anti-inflammatory properties (Hileman and Funderburg, 2017), but drugs of abuse may interact pharmacologically with ART to reduce its effectiveness in both suppressing viral load and minimizing cognitive deficits associated with HAND (Kumar et al., 2015). Future studies should examine the efficacy of anti-inflammatory adjunctive therapeutics in reducing HIV-associated neurocognitive deficits and promoting long-term abstinence from drug use.

## Conclusion

Throughout this review, we have highlighted recent studies that demonstrate functionally significant immunomodulatory mechanisms that may underlie the pathophysiology of SUDs and associated comorbidities. While immunomodulation of learning, memory, and synaptic plasticity is not a new concept *per se*, recent research has greatly expounded the role of immune signaling in neuropsychiatric disorders and diseases, providing a firm foundation for future investigations into novel treatment targets. The literature presented herein suggests that central and peripheral immune mechanisms may represent a common thread underlying the interactions between SUDs and associated comorbidities. Indeed, the clinical and preclinical findings discussed here underscore the need for future investigations into the efficacy of adjunctive immunomodulatory medications that may be therapeutically efficacious in the treatment of SUDs and associated comorbidities such as MDD, PTSD, chronic pain, and HIV. Despite these advances, one major gap in the literature that demands future investigation is how neuroimmune signaling within mesocorticolimbic circuits is altered across the entire addiction cycle. For example, very few studies to date have examined how neuroimmune signaling is altered across a protracted period of abstinence compared to acute and chronic drug exposure, and if such changes are causally linked to drug-seeking behavior. Moreover, very few studies to date have thoroughly examined how comorbidities impact neuroimmune function during periods of active drug taking versus periods of protracted abstinence. Abstinence represents a critical period of vulnerability within the addiction cycle, and the neuroimmune sequelae of chronic drug use and abstinence remains poorly understood. Investigation of these processes could improve our understanding of how motivation for drug increases (or “incubates”) over time throughout a protracted period of abstinence to increase relapse vulnerability (Pickens et al., 2011). As mentioned numerous times throughout this review, the role of sex differences in the neuroimmune mechanisms of SUDs and associated comorbidities remains poorly understood. Indeed, there are significant sex differences in the prevalence rates of individual conditions such as SUDs, MDD, PTSD, chronic pain, etc. As thoroughly reviewed by others (Rosen et al., 2017; Bekhbat and Neigh, 2018; Osborne et al., 2018; McCarthy, 2019), there are sex-specific neuroimmune mechanisms involved in the pathophysiology of these conditions that may be suitable targets for medications development. Nevertheless, whether these sex-specific neuroimmune mechanisms drive the comorbidity of these disorders and diseases and contribute to their disparate prevalence rates is largely unknown and future research is needed to address this gap. Many of the studies discussed herein are largely observational in nature and few have attempted to experimentally manipulate neuroimmune mechanisms using drug self-administration animal models, which is another remaining gap in the field. For example, there are significant differences in the neurobiological mechanisms underlying cue-induced drug-seeking behavior between animal models involving experimenter-delivered drug versus self-administration of drug (Namba et al., 2018), and these mechanisms may be differentially regulated by distinct neuroimmune mechanisms. Overall, we suggest that future research needs to address the gaps mentioned above to better assess whether putative neuroimmune targets are suitable candidates for medications development to treat SUDs and associated comorbidities.

## Author Contributions

MN conceptualized this review. MN and JL-J wrote the first draft. EN, MO, and JN subsequently contributed to the content, ideas, and editing of the manuscript. All authors contributed to the article and approved the submitted version.

## Conflict of Interest

The authors declare that the research was conducted in the absence of any commercial or financial relationships that could be construed as a potential conflict of interest.
